# Clinicopathological Implications of IDH1 Mutation and Ki-67 Expression Across WHO Grades of Gliomas: Insights from a Prospective Observational Study

**DOI:** 10.22034/ijp.2026.2072578.3624

**Published:** 2026-08-01

**Authors:** Khushboo Sinha, Sudha Iyengar, Reema Bhushan, Avinash Sharma

**Affiliations:** 1 Department of Pathology, Gajra Raja Medical College and Hospital, Yadav Colony, Lashkar, Gwalior, Madhya Pradesh, India; 2 Department of Neurosurgery, Gajra Raja Medical College, Gwalior, Madhya Pradesh, India

**Keywords:** Glioblastoma, isocitrate dehydrogenase, IDH1 R132H, astrocytoma

## Abstract

**Background & Objective:**

Gliomas are heterogeneous central nervous system tumors with variable outcomes. Accurate classification using histopathological and molecular markers, such as IDH1 R132H mutation and Ki-67 index, is essential. This study evaluated their distribution across glioma grades and correlation with clinicopathological parameters.

**Methods:**

This prospective observational study included 90 histologically confirmed glioma cases. Hematoxylin and eosin (H&E)-stained sections were graded according to WHO 2021 criteria. Immunohistochemistry was used to assess IDH1 R132H mutation status and Ki-67 labeling index. Associations between variables were analyzed using chi-square and logistic regression.

**Results:**

Astrocytomas (55/90, 61.1%) and glioblastomas (24/90, 26.7%) were the most common tumor types. IDH1 R132H mutation was detected in 65/90 cases (72.2%). Grade-wise, mutation positivity was highest in grade II (28/28, 100%), followed by grade I (8/9, 88.9%) and grade III (20/26, 76.9%), whereas grade IV glioblastomas showed significantly lower positivity (9/27, 33.3%). Ki-67 expression increased with tumor grade, with >20% expression in 26/27 grade IV tumors (96.3%). A significant inverse correlation was observed between IDH1 mutation status and Ki-67 level (P < 0.0001). Logistic regression showed that younger age was significantly associated with IDH1 mutation (P = 0.001), while IDH1 wild-type status correlated with older age (P = 0.024).

**Conclusion:**

IDH1 R132H mutation and Ki-67 index correlate with glioma grade and age, supporting their routine use in classification. In the absence of survival data, the present results reflect clinicopathological associations rather than validated prognostic predictions; prospective studies incorporating survival outcomes are needed to confirm the prognostic utility of these markers.

## Introduction

Gliomas constitute a significant proportion of central nervous system (CNS) tumors, representing approximately 30% of primary brain tumors and 80% of malignant CNS tumors(1–3). These tumors mostly impact the adult population, with glioblastoma multiforme (GBM), the most aggressive subtype, accounting for nearly half of glioma cases worldwide(3). The incidence of GBM is approximately 3.2 cases per 100,000 person-years, making it one of the most challenging conditions in neuro-oncology due to its aggressive nature and poor prognosis(3–5). Other noteworthy subtypes, such as astrocytomas, oligodendrogliomas, and ependymomas, show distinct demographic profiles, clinical presentations, and prognostic outcomes, collectively contributing to the complexity of glioma epidemiology(6). Gliomas occur most commonly in individuals aged between 45 and 65 years(4). Low-grade astrocytomas primarily occur among younger adults aged 20 to 40 years, while high-grade astrocytomas, including anaplastic astrocytomas and glioblastomas, occur among middle-aged to older adults(7,8). The epidemiological results indicate a marked gender difference, in which cases are more common among males than females, with a male-to-female ratio of approximately 1.4-1.5:1, further highlighting the importance of demographic factors in the investigation of gliomas and therapy options(9,10).

The World Health Organization (WHO) central nervous system (CNS) tumor classification system has undergone significant developments, with the latest version being released in 2021(11,12). This updated classification integrates histopathological assessment with advanced molecular diagnostics, substantially improving diagnostic precision(13). An essential molecular marker recognized by the WHO is the isocitrate dehydrogenase (IDH) mutation, specifically the IDH1 R132H mutation(14). This mutation is highly prevalent in lower-grade gliomas and secondary glioblastomas. It is associated with improved patient outcomes, with increased overall and progression-free survival. The mutations are hypothesized to be among the earliest genetic changes in gliomagenesis, thus cementing their pivotal position in tumor biology and their therapeutic potential as targets(13,14). Mechanistically, IDH mutations lead to the abnormal production of 2-hydroxyglutaric acid, a metabolite that inhibits alpha-ketoglutarate-dependent dioxygenases, affecting DNA demethylation and histone modification processes(15,16). Thus, these epigenetic variations result in widespread hypermethylation, encouraging tumorigenesis and affecting the differentiation potential of glioma stem cells(15). Moreover, IDH mutation status helps differentiate primary glioblastomas, typically IDH-wildtype and presenting de novo, from secondary glioblastomas, predominantly IDH-mutant and arising from the progression of lower-grade gliomas(15,16). Such distinction has important clinical implications for prognosis and treatment planning.

Ki-67, a nonhistone nuclear protein that is expressed in the active phases of the cell cycle, is primarily employed as a proliferation marker in oncology(17). In glioma research, Ki-67 expression is correlated with tumor grade, cellular proliferation, and clinical outcome. High Ki-67 indices typically indicate aggressive tumor behaviour and unfavorable prognoses, whereas low indices indicate less aggressive tumor biology. Integrated assessment of IDH mutation status and Ki-67 expression enhances diagnostic accuracy, with important information regarding tumor aggressiveness, infiltration borders, and response to treatments(17,18). With the development of glioma diagnostics, however, certain knowledge gaps in glioma biology persist, such as IDH mutation frequency heterogeneity among populations and among glioma subtypes. The clinical significance of IDH mutations by tumor location, patient age, and histological characteristics is still poorly demarcated(19). Bridging the gaps through large-scale, population-based research can add significantly to the adaptation of therapeutic approaches and improved patient management.

This study aims to address these research gaps by investigating the frequency, distribution, and clinical correlations of IDH1 R132H mutations in gliomas across different WHO grades within a tertiary care setting. Additionally, the research evaluates the association between IDH mutation status and Ki-67 expression. Through systematic analysis of the histopathological and molecular attributes of gliomas, the study aims to delineate clinicopathological associations, contribute to diagnostic precision, and provide a foundation for future studies assessing the therapeutic and prognostic implications of these markers.

## Materials and Methods

### Study Setting

The study was conducted in the Department of Pathology, Gajra Raja Medical College and Hospital, Gwalior, Madhya Pradesh, India, a teaching tertiary care hospital with specialized neurosurgical care and advanced pathological investigation. The institutional ethics committee approved the study protocol, and the study was conducted following ethical practices.

### Study Design

This study was designed as a prospective observational study. Cases were enrolled consecutively over a 2-year period, with each patient assessed at a single time point corresponding to the diagnostic workup of the surgical specimen. This design enabled systematic characterization of histopathological features and immunohistochemical patterns, including IDH1 R132H mutation status and Ki-67 proliferation index, across newly diagnosed glioma cases. The 2-year enrollment window provided an adequate sample size representative of the glioma pathology encountered at this tertiary care center. No formal a priori sample size calculation was performed; however, the cohort of 90 cases provided sufficient representation across all 4 WHO grades to permit meaningful statistical comparisons.

### Selection Criteria

The inclusion criteria were all surgical resections and biopsy tissue received in the Department of Pathology and histologically diagnosed as gliomas according to the WHO 2021 classification. All the material was collected from the Department of Neurosurgery, where clinical and radiological study data were well documented. The exclusion criteria were cases with insufficient tissue material, poorly documented clinical data, prior chemotherapy or radiotherapy, or incomplete histopathological examination that would mislead accurate diagnostic classification and molecular profiling.

### Sample Processing and Histopathological Analysis

Received glioma tissues were initially fixed in 10% buffered formalin for approximately 24–48 hours following standard histological protocols. The tissue samples were dehydrated in graded alcohol, cleared in xylene, and embedded in paraffin blocks. Serial sections of 4–5 µm thickness were subsequently cut with a rotary microtome. Paraffin-embedded sections were stained with hematoxylin and eosin (H&E) for initial histopathological assessment. Microscopic assessment involved meticulous observation of cellular morphology of the tumor, nuclear atypia, mitotic figures, microvascular proliferation, and necrosis. Special grading of each case was made with reference to the WHO 2021 guidelines, with both histological appearance and molecular characteristics in mind, wherever available.

### Immunohistochemical Analysis

Immunohistochemical (IHC) analysis was performed to ascertain IDH1 R132H mutation status and Ki-67 expression levels. Briefly, paraffin-embedded tissue sections were mounted onto positively charged slides and subjected to heat-induced antigen retrieval in citrate buffer (pH 6.0) using a microwave oven, enhancing antigen exposure for antibody binding. Following antigen retrieval, sections were treated to block endogenous peroxidase activity using 3% hydrogen peroxide solution. Subsequently, primary antibodies targeting IDH1 R132H mutant protein (clone H09, Dianova GmbH, Germany) IDH1 wild-type protein (clone IDH1/1152), and Ki-67 (clone MIB-1, Dako, Denmark) were applied at dilutions recommended by the manufacturer and incubated overnight at 4 °C in a humidified chamber. After thorough washing with phosphate-buffered saline (PBS), sections were incubated with biotinylated secondary antibodies, followed by avidin-biotin-peroxidase complex solution (Vectastain Elite ABC kit, Vector Laboratories). Visualization of antigen-antibody complexes was achieved using 3,3′-diaminobenzidine (DAB) as the chromogenic substrate, yielding a brown precipitate at the site of antigen presence. Sections were counterstained lightly with hematoxylin to enhance nuclear detail visualization. Appropriate positive and negative controls were included in each staining batch to validate the reliability of the staining procedure.

### Evaluation of Immunohistochemical Staining

The evaluation of IDH1 R132H immunostaining was based on cytoplasmic positivity within tumor cells. Cases demonstrating unequivocal brown cytoplasmic staining were considered positive for IDH R132H mutation. IDH1 wild-type immunostaining (clone IDH1/1152) was evaluated independently, based on cytoplasmic positivity within tumor cells, and scored as positive or negative for wild-type protein expression; IDH1 R132H mutant status and IDH1 wild-type status were thus assessed as two separate, antibody-specific stains rather than one being inferred from the other, which accounts for cases in which both stains were scored positive (Table 1). Ki-67 staining was assessed by calculating the labeling index, defined as the percentage of positively stained tumor nuclei out of a total of 1000 counted tumor cells under high-power fields (400× magnification). Ki-67 indices were categorized as low (<3%), moderate (3%–20%), and high (>20%) to correlate with tumor aggressiveness and prognosis.

**Table 1:** Expression of IDH 1R132H mutation, wild type, and Ki-67 according to WHO grading.

			WHO GRADE				Total
1	2	3	4
IDH1 R132H mutation	Negative	N	1	0	6	18	25
		%	11.1%	0.0%	23.1%	66.7%	27.8%
	Positive	N	8	28	20	9	65
		%	88.9%	100.0%	76.9%	33.3%	72.2%
Total		N	9	28	26	27	90
		%	100.0%	100.0%	100.0%	100.0%	100.0%
Chi-square :32.656; df:3; p=<0.0001							
IDH 1 Wild type	Negative	N	9	27	14	8	58
		%	100.0%	96.4%	53.8%	29.6%	64.4%
	Positive	N	0	1	12	19	32
		%	0.0%	3.6%	46.2%	70.4%	35.6%
Total		N	9	28	26	27	90
		%	100.0%	100.0%	100.0%	100.0%	100.0%
Chi-square :33.023; df:3; p= <0.0001							
Ki-67 Expression	<3%	N	9	8	0	0	17
		%	100.0%	28.6%	0%	0%	18.9%
	3 -20%	N	0	20	15	1	36
		%	0.0%	71.4%	57.7%	3.7%	40.0%
	>20%	N	0	0	11	26	37
		%	0.0%	0.0%	42.3%	96.3%	41.1%
Total		N	9	28	26	27	90
		%	100.0%	100.0%	100.0%	100.0%	100.0%
Chi-square :99.410; df:6; p=<0.0001							

### Statistical Analysis

Collected data were entered into a structured proforma and statistically analyzed using SPSS software version 26.0. Descriptive statistics were applied to summarize the demographic data, histological types, tumor grades, and immunohistochemical findings, expressed in frequencies, percentages, means, and standard deviations (SDs). Categorical variables, including tumor grade, IDH mutation status, Ki-67 labeling index, and clinical characteristics, were analyzed using chi-square tests to assess potential associations. Continuous variables, such as patient age, were examined using independent samples t tests or Mann-Whitney U tests, depending on the data distribution’s normality, which was assessed using the Kolmogorov-Smirnov test. The significance level for all statistical tests was established at a P value of less than 0.05. The statistical analysis aimed to delineate correlations between IDH1 mutation status, Ki-67 index, glioma grades, and various demographic and clinical parameters, providing insights into prognostic and therapeutic implications.

### Quality Control

Stringent quality control measures were adhered to throughout the study. Interobserver variability in histological grading and immunohistochemical interpretation was minimized by involving multiple experienced pathologists who independently assessed and subsequently discussed each case to reach a consensus diagnosis. Additionally, regular calibration and maintenance of laboratory equipment ensured consistency and reliability of results.

All patient data and tissue samples were handled confidentially, adhering strictly to ethical standards and institutional guidelines. The study received approval from the Institutional Ethical Committee, and informed consent was obtained from all participants or their legal representatives prior to specimen collection and analysis, ensuring full compliance with ethical research standards. Through this meticulous methodological approach, the study aimed to provide robust and clinically meaningful insights into the histopathological and molecular characteristics of gliomas, potentially enhancing diagnostic accuracy and informing personalized therapeutic strategies.

## Results

**Table 2:** Age group distribution according to frequency of glioma.

Age group distribution			Gender Distribution		
Age group (Years)	Frequency	Percentage	Gender	Frequency	Percentage
≤ 20	6	6.7	Female	33	36.7
21-30	22	24.4	Male	57	63.3
31-40	13	14.4	WHO grade distribution		
41-50	17	18.9	Grade I	9	10.0
51-60	26	28.9	Grade II	28	31.1
61-70	3	3.3	Grade III	26	28.9
>70	3	3.3	Grade IV	27	30.0
Frequency of IDH1R132H mutation			Frequency of IDH wild type		
Negative	25	27.8	Negative	58	64.44
Positive	65	72.2	Positive	32	35.56
Ki-67 expression status			Location wise distribution		
<3 %	17	18.89	Frontal	42	46.7
>3-<20%	36	40	Parietal	15	16.7
>20%	37	41.11	Temporal	33	36.7
Laterality of the cases			Histopathological diagnosis		
Bilateral	1	1.1	Astrocytoma	55	61.1
Left	36	40	Ependymoma	1	1.1
Right	52	57.8	Glioblastoma	24	26.7
Posterior (4th ventricle)	1	1.1	Pilocytic Astrocytoma	6	6.7
Total	90	100	Oligodendroglioma	4	4.4

### Patient Demographics and Clinical Characteristics (Table 2)

**Fig. 1:**
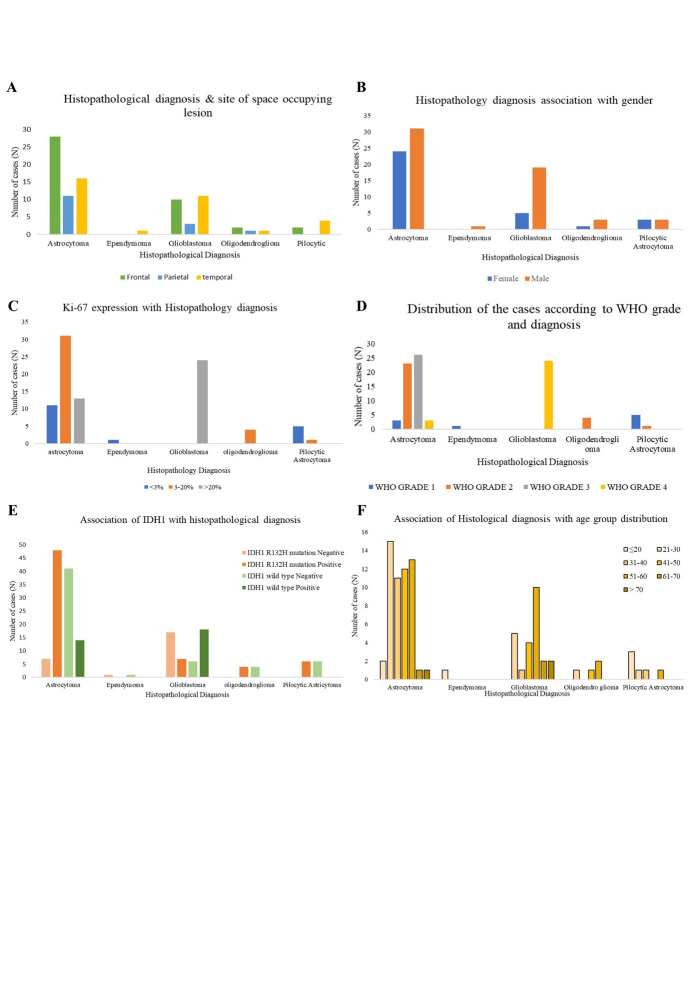
Distribution of glioma cases by clinicopathological and molecular parameters. (A) Anatomical distribution of space-occupying lesions across histopathological types, with frontal localization most common in astrocytomas and glioblastomas. (B) Gender-wise distribution reveals male predominance across all glioma subtypes. (C) Ki-67 expression stratified by histological diagnosis shows higher proliferation indices in glioblastomas. (D) WHO grade distribution across tumor types indicates that astrocytomas span multiple grades, while glioblastomas are exclusively grade IV. (E) IDH1 mutation and wild-type expression by histological type showing highest IDH1 R132H positivity in astrocytomas and oligodendrogliomas; glioblastomas are largely wild-type. (F) Age-group distribution across histological types indicates younger age prevalence in astrocytomas and older age in glioblastomas.

Among the 90 glioma cases, the highest age group distribution was in the 51–60-year age range (28.9%), followed by the 21–30-year age range (24.4%), and a male predominance was observed (63.3%). WHO grade II tumors were the most frequent (31.1%), followed closely by grade III (28.9%) and grade IV (30%), with grade I accounting for 10%. IDH1 R132H mutation was positive in 72.2% of cases (IDH1 R132H-positive); IDH1 wild-type immunoreactivity was separately identified in 35.56% of cases. Ki-67 expression was low (<3%) in 18.9%, moderate (3%–20%) in 40% and high (>20%) in 41.1% of cases, indicating a wide range of proliferative activity. The frontal lobe was the most common tumor location (46.7%), followed by the temporal lobe (36.7%) and the parietal lobe (16.7%). Tumors were predominantly right-sided (57.8%), with 40% located on the left, 1.1% bilateral, and 1.1% in the fourth ventricle. Astrocytomas accounted for the majority (61.1%), followed by glioblastomas (26.7%), pilocytic astrocytomas (6.7%), oligodendrogliomas (4.4%), and a single case of ependymoma (1.1%) (Fig. 1).

### Clinicopathological and Molecular Correlations in Glioma Cases

**Fig. 2:**
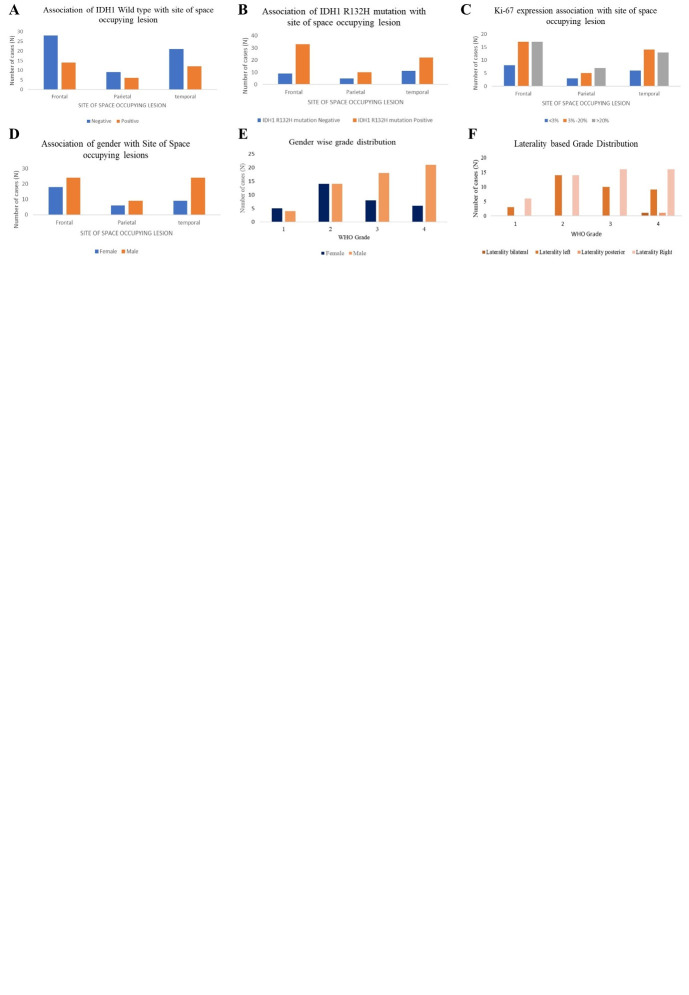
Association of IDH1 status, Ki-67 index, sex, and tumor site with clinical and anatomical parameters. (A and B) IDH1 wild-type and R132H mutation distribution across the frontal, parietal, and temporal lobes. (C) Ki-67 expression by tumor site reveals the highest proliferation in temporal lobe tumors. (D) Sex distribution by site shows male predominance, particularly in frontal and temporal tumors. (E) Grade-wise sex distribution indicates male predominance in higher grades. (F) Tumor laterality by grade shows right-sided dominance, especially in grade IV tumors. (G) Age-wise distribution of tumor site highlights peak occurrence of frontal lesions in the 51–60-year age group. (H) WHO grade-wise site distribution shows frontal lobe predominance in grades II–IV.

Astrocytomas were predominantly located in the frontal lobe, while glioblastomas were more evenly distributed between the frontal and temporal lobes (Fig. 1A). Male predominance was noted across all histological subtypes, particularly in astrocytomas and glioblastomas (Fig. 1B). Ki-67 proliferation indices varied significantly by tumor type; astrocytomas mostly exhibited moderate expression (3%–20%), whereas glioblastomas showed high proliferative activity, with Ki-67 >20% in most cases (Fig. 1C). WHO grade distribution revealed that astrocytomas spanned grades I to III, glioblastomas corresponded exclusively to grade IV, and pilocytic astrocytomas were confined to grade I (Fig. 1D). IDH1 R132H mutation was most frequently positive in astrocytomas and oligodendrogliomas, while glioblastomas were predominantly IDH1 wild-type (Fig. 1E). Age-wise analysis revealed that astrocytomas were distributed across all age groups but were most common in individuals aged 21–50 years. In contrast, glioblastomas occurred most frequently in the 51–60-year age group (Fig. 1F). These findings indicate distinct molecular and clinical profiles among glioma subtypes, with IDH1 mutation and Ki-67 index showing significant associations with tumor type and grade (Fig. 2).

### Molecular, Anatomical, and Demographic Associations with Site and Grade of Gliomas

**Fig. 3:**
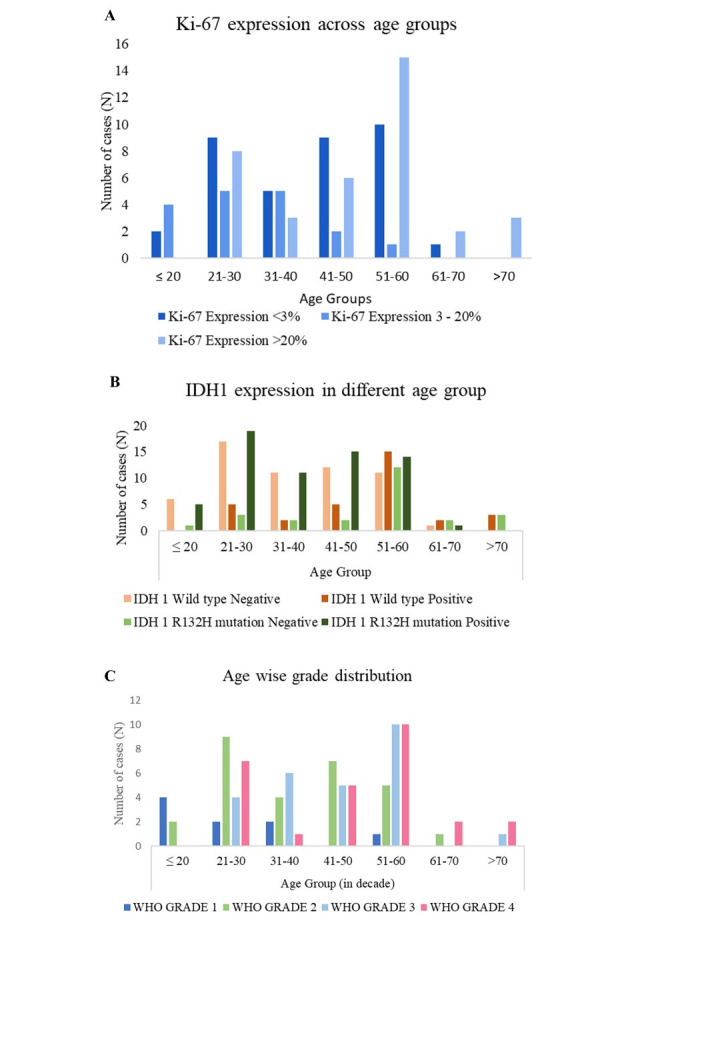
Age-stratified expression of Ki-67, IDH1, and WHO grade. (A) Ki-67 expression shows a higher proliferative index (>20%) predominantly in the 51–60-year age group. (B) IDH1 R132H mutation is more prevalent in younger individuals aged 21–50 years, while wild-type expression increases with age. (C) WHO grade distribution across age groups shows lower grades (I and II) in younger patients and higher grades (III and IV) in older age groups.

IDH1 wild-type expression was more frequent in frontal lobe lesions, while its positivity declined in temporal and parietal locations (Fig. 2A). In contrast, IDH1 R132H mutation positivity was highest in frontal and temporal regions, indicating a predilection for these sites among mutated tumors (Fig. 2B). Ki-67 proliferation indices showed a broad distribution across sites, with high expression (>20%) being most frequent in temporal lobe tumors (Fig. 2C). Frontal tumors were more common in males, while temporal lesions showed near-equal sex distribution (Fig. 2D). Grade-wise analysis showed male predominance in higher WHO grades (III and IV), while females were more frequent in lower grades (Fig. 2E). Right-sided gliomas were most common across all grades, with grade IV tumors being strongly right-lateralized (Fig. 2F). Age-wise distribution revealed that frontal lobe tumors peaked in the 51–60-year group, while parietal and temporal tumors were more evenly distributed across decades (Supplementary Fig. 1A). Grade-wise site distribution showed that frontal lobe lesions were predominant across all WHO grades, with the highest number in grade III tumors (Supplementary Fig. 1B). These patterns emphasize distinct anatomical preferences and demographic trends across glioma grades and molecular subtypes (Fig. 3).

Age-Related Variations in Ki-67 Expression, IDH1 Mutation, and Tumor Grade in Gliomas

Ki-67 expression patterns showed age-dependent variation, with low (<3%) and moderate (3%–20%) indices most frequent in the 21–30- and 51–60-year age groups, while high proliferation rates (>20%) were predominantly observed in the 51–60-year age group, indicating increased tumor aggressiveness with advancing age (Fig. 3A). IDH1 wild-type negativity and R132H mutation positivity were most common between 21 and 60 years, particularly in the 21–30- and 51–60-year age brackets, while mutation-negative and wild-type-positive cases were rare in older individuals, suggesting that younger age groups are more likely to harbor favorable molecular profiles (Fig. 3B). Grade-wise age distribution demonstrated that lower-grade gliomas (WHO grades I and II) were more frequent in younger patients (≤30 years), whereas higher-grade tumors (grades III and IV) increased in frequency with age, peaking between 41 and 60 years (Fig. 3C). These findings highlight distinct age-related trends in glioma biology, with younger patients showing a predominance of IDH1-mutant, lower-grade tumors and lower Ki-67 indices, while older patients more often presented with high-grade, highly proliferative, IDH1 wild-type gliomas.

Binary logistic regression analysis was performed to evaluate the predictive influence of age and sex on IDH1 R132H mutation and IDH1 wild-type expression. For IDH1 R132H mutation, age emerged as a statistically significant negative predictor (B = –0.081, P = 0.001), indicating that with increasing age, the likelihood of harboring the mutation decreased (Exp[B] = 0.923; 95% CI, 0.879–0.968). Sex was not significantly associated with IDH1 mutation status (P = 0.152), though male sex showed a lower odds ratio (OR = 0.335). In contrast, for IDH1 wild-type expression, age was a significant positive predictor (B = 0.169, P = 0.024), suggesting an increased likelihood of wild-type status with advancing age (Exp[B] = 1.184; 95% CI, 1.022–1.371). Sex again was not a significant factor (P = 0.382). These findings suggest that age is an independent and opposing predictor of IDH1 molecular status, favoring mutation in younger individuals and wild-type expression in older ones, whereas sex did not show a significant impact in either model (Table 3, Table 1). Association of WHO Tumor Grade With IDH1 R132H Mutation, IDH1 Wild-Type Status, and Ki-67 Expression

**Table 3:** Variables (age and sex) in IDH1 R132H mutation using logistic regression formula

Variables in the Equation						
		B	Sig.	Exp(B)	95% C.I. for EXP(B)	
					Lower	Upper
IDH1 R132H mutation	AGE	-.081	.001	.923	.879	.968
	SEX	-1.094	.152	.335	.075	1.496
	Constant	5.841	.000	344.259		
IDH1 Wild type	AGE	.169	.024	1.184	1.022	1.371
	SEX	-1.293	.382	.274	.015	4.993
	Constant	-3.493	.148	.030		
logistic regression is applied using the Variable(s) entered on step 1: AGE, SEX.						

IDH1 R132H mutation was most prevalent in lower-grade gliomas, with 100% positivity in grade II and 76.9% in grade III tumors. In contrast, grade IV gliomas showed a substantial decline in mutation positivity (33.3%), while 66.7% of mutation-negative cases were grade IV. This association was statistically significant (χ² = 32.656; P < 0.0001). Regarding IDH1 wild-type status, negativity was more common in lower grades (100% in grade I and 96.4% in grade II), whereas positivity sharply increased with grade, reaching 70.4% in grade IV, confirming a strong correlation (χ² = 33.023; P < 0.0001). Ki-67 expression showed a clear upward trend with increasing grade. All grade I tumors had low Ki-67 indices (<3%), while 71.4% of grade II tumors showed moderate expression (3%–20%). High Ki-67 expression (>20%) was strongly associated with grade III (42.3%) and grade IV (96.3%) tumors. The association between Ki-67 expression and tumor grade was highly significant (χ² = 99.410; P < 0.0001), supporting its role as a marker of tumor proliferation and aggressiveness. Collectively, these data demonstrate that IDH1 mutation positivity and low Ki-67 index are linked with lower tumor grades, whereas IDH1 wild-type expression and high Ki-67 levels correlate strongly with higher-grade gliomas.

### Histopathological and Immunohistochemical Features Across Various Grades and Subtypes of Gliomas

**Fig. 4:**
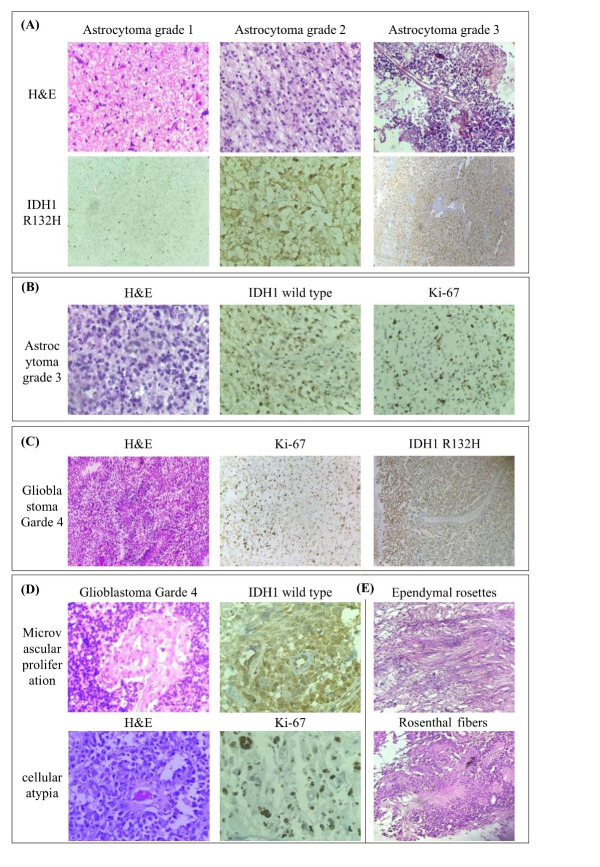
Representative histological and immunohistochemical features of glioma subtypes. (A) Astrocytoma grades I–III show increasing cellularity and IDH1 R132H positivity with grade progression. (B) Grade III astrocytoma exhibiting nuclear Ki-67 positivity and IDH1 wild-type expression. (C) Glioblastoma (grade IV) showing pseudopalisading necrosis (H&E), high Ki-67 index, and focal IDH1 R132H positivity. (D) High-grade glioblastoma with microvascular proliferation, cellular atypia, IDH1 wild-type expression, and elevated Ki-67. (E) Low-grade gliomas: ependymoma with true rosettes and pilocytic astrocytoma with Rosenthal fibers and cystic areas.

Histopathological and immunohistochemical findings in this study revealed consistent morphological features corresponding with tumor grade and molecular status. Low-grade astrocytomas (grades I and II) displayed low to moderate cellularity with minimal atypia, accompanied by strong IDH1 R132H mutation positivity, whereas grade III astrocytomas showed increased cellularity and perivascular proliferation with sustained IDH1 expression and rising Ki-67 indices, indicating progression in proliferative activity (Fig. 4A). Grade III astrocytomas that were IDH1 wild-type demonstrated more atypical cell morphology and elevated Ki-67 expression, suggesting more aggressive biological behavior even within the same histological category (Fig. 4B). Glioblastomas (grade IV) consistently exhibited hallmark features such as pseudopalisading necrosis, microvascular proliferation, and marked cellular atypia. These cases were predominantly IDH1 wild-type and showed strong Ki-67 nuclear staining, reinforcing their high-grade nature and proliferative potential (Fig. 4C and 4D). In contrast, low-grade tumors such as ependymomas and pilocytic astrocytomas retained characteristic features like ependymal rosettes and Rosenthal fibers with cystic changes, lacking aggressive proliferation patterns (Fig. 4E). These findings support the observed associations between histological grade, IDH1 mutation status, and Ki-67 index, affirming their diagnostic and prognostic relevance (Fig. 4).

## Discussion

This prospective study evaluated 90 cases of histologically confirmed gliomas to explore the relationship between IDH1 R132H mutation status, Ki-67 proliferation index, tumor grade, anatomical site, and demographic variables. The findings reinforce the emerging consensus that molecular profiling adds significant prognostic and diagnostic value to traditional histopathological assessment. In this cohort, astrocytomas represented the most prevalent subtype, followed by glioblastomas, pilocytic astrocytomas, oligodendrogliomas, and a single case of ependymoma. This distribution aligns with global epidemiological patterns and the classification criteria outlined in the World Health Organization (WHO) 2021 guidelines, which emphasize the need for integrated histomolecular diagnosis of gliomas(20).

A slight male predominance and a peak incidence in the 51–60-year age group were observed, consistent with previous studies showing that glioblastoma multiforme (GBM) and other high-grade gliomas are more common in males and typically present in middle to late adulthood(21,22). The age distribution in this study also demonstrated that lower-grade gliomas occurred more frequently in younger patients, whereas higher-grade gliomas, particularly glioblastomas, were predominant in older age groups, supporting the age-stratified trends reported by the Cancer Genome Atlas Research Network (2018) and others(23,24). The IDH1 R132H mutation was detected in 72.2% of all cases, with significantly higher positivity in lower-grade tumors. All grade II astrocytomas and 76.9% of grade III astrocytomas were IDH1-mutant, while only 33.3% of glioblastomas exhibited the mutation. These findings are consistent with previous studies that have described the IDH1 mutation as a hallmark of lower-grade gliomas and secondary glioblastomas arising from pre-existing low-grade tumors(23,25). The mutation is recognized as one of the earliest events in gliomagenesis, and its presence correlates with a more favorable prognosis and longer survival, often exceeding 5 years compared to 12–15 months in IDH1 wild-type glioblastomas(26,27).

In addition to being a favorable prognostic indicator, IDH1 status has emerged as a key therapeutic biomarker. A recent phase III clinical trial (INDIGO) demonstrated that vorasidenib, a dual IDH1/2 inhibitor, significantly delayed disease progression in patients with IDH-mutant low-grade gliomas, supporting the therapeutic relevance of identifying this mutation(28). In our analysis, logistic regression further confirmed that younger age significantly predicted IDH1 mutation positivity, whereas older patients were more likely to harbor IDH1 wild-type gliomas. This inverse age-mutation relationship has also been reported in previous multicentric studies and has implications for screening and treatment planning(10,29). Ki-67, a nuclear marker of cellular proliferation, showed a clear correlation with WHO grade in this cohort. Low Ki-67 index (<3%) was confined to grade I and a minority of grade II tumors, while high Ki-67 expression (>20%) was predominant in more than 96% of glioblastomas. These findings mirror existing reports demonstrating a direct relationship between Ki-67 labeling index and tumor aggressiveness. In one large-scale meta-analysis, Ki-67 was shown to correlate strongly with progression-free survival in gliomas and served as an independent prognostic indicator, particularly in high-grade tumors(30,31).

An important observation in this study was the inverse relationship between IDH1 mutation status and Ki-67 expression. IDH1-mutant tumors generally exhibited lower Ki-67 indices, whereas IDH1 wild-type gliomas showed markedly higher proliferative activity. This trend supports earlier studies where dual stratification using IDH1 and Ki-67 yielded better prognostic categorization than either marker alone(31). The biological rationale behind this relationship is rooted in the role of mutant IDH1 in altering cellular metabolism, promoting a less proliferative but more differentiated tumor phenotype(31,32). Spatial distribution of tumors revealed that frontal lobe involvement was most common across histological subtypes and grades, particularly in astrocytomas and glioblastomas. Right-sided tumors were more frequent in grade IV cases. These observations align with previously reported anatomical preferences of gliomas, where high-grade tumors tend to arise in the cerebral hemispheres, predominantly in frontal and temporal regions(33). Age- and location-related trends in our study also corroborate findings from spatial modeling of glioma incidence, which suggest that younger patients more commonly develop low-grade tumors in posterior fossa or cerebellar regions, while older patients develop more aggressive tumors in the cerebral hemispheres(21,26).

The histopathological findings in this cohort supported the WHO 2021 grading system, with grade I astrocytomas demonstrating low cellularity and minimal atypia and grade IV glioblastomas showing marked nuclear pleomorphism, microvascular proliferation, and pseudopalisading necrosis. Immunohistochemical staining further validated the histological grading, with high Ki-67 labeling and IDH1 wild-type expression aligning with grade IV features, while IDH1-mutant, low Ki-67 tumors corresponded to low-grade morphology (Fig. 4). Classical features such as Rosenthal fibers in pilocytic astrocytomas and true ependymal rosettes in ependymomas were clearly observed, strengthening the morphological diagnoses. These findings reinforce the importance of an integrated diagnostic approach combining histopathological evaluation with molecular markers. The inclusion of IDH1 status and Ki-67 index significantly enhances the prognostic precision of glioma classification. This integrated framework aligns with the WHO 2021 revision, which emphasizes molecular profiling not only for diagnosis but also for treatment stratification and clinical trial eligibility(24,25). Studies have shown that the combination of IDH1 mutation and low Ki-67 is associated with the longest survival, while IDH1 wild-type tumors with high Ki-67 have the worst outcomes(30,31).

Despite the strengths of this study, including its prospective design and standardized immunohistochemical analysis, certain limitations warrant consideration. First, the single-center design limits the generalizability of these findings to other institutional or geographic settings, and a referral bias toward higher-grade tumors cannot be excluded. Second, IDH1 R132H immunohistochemistry, while sensitive for the common R132H variant, does not detect the full spectrum of IDH1 and IDH2 mutations; molecular sequencing or next-generation panel testing would be required for comprehensive IDH status assessment, particularly in cases with equivocal or negative IHC in clinically low-grade tumors. Third, Ki-67 labeling index assessment by manual counting is semiquantitative and subject to interobserver variability and field selection bias; digital image analysis would improve reproducibility. Fourth, the study did not include additional molecular markers integral to the WHO 2021 classification, such as 1p/19q codeletion, MGMT promoter methylation, ATRX loss, and TERT promoter mutations, which would allow more complete molecular stratification, particularly in IDH-wild-type cases with an indolent clinical course. Fifth, and most critically, no survival or outcome data were collected; therefore, all conclusions regarding prognostic implications are associative only and cannot be extrapolated to predict individual patient outcomes. Future studies incorporating longitudinal follow-up, survival analysis, and broader molecular profiling in multicenter cohorts are required to validate and extend these findings(34,35).

## Conclusion

In conclusion, the results of this prospective study demonstrate significant associations between IDH1 R132H mutation status, Ki-67 proliferation index, and glioma grade, patient age, and anatomical location. These findings support the inclusion of IDH1 R132H immunohistochemistry and Ki-67 assessment in routine diagnostic workflows for glioma classification. However, in the absence of survival analysis or longitudinal follow-up, the current data do not permit conclusions regarding prognostic accuracy or clinical decision-making utility, and caution is warranted in overstating the predictive value of these markers. As IDH1-targeted therapies gain traction in clinical practice, prospective identification of IDH1 mutation status at diagnosis may increasingly become essential. Further multicenter studies incorporating broader molecular panels and survival data will be necessary to fully translate these findings into clinical practice.

## Data Availability

The data that support the findings of this study are available from the corresponding author upon request.
